# Clinical impact of early response to first‐line VEGFR‐TKI in patients with metastatic renal cell carcinoma on survival: A multi‐institutional retrospective study

**DOI:** 10.1002/cam4.5268

**Published:** 2022-10-06

**Authors:** Ryuta Sobu, Kazuyuki Numakura, Sei Naito, Shingo Hatakeyama, Renpei Kato, Tomoyuki Koguchi, Takahiro Kojima, Yoshihide Kawasaki, Syuya Kandori, Sadafumi Kawamura, Yoichi Arai, Akihiro Ito, Hiroyuki Nishiyama, Yoshiyuki Kojima, Wataru Obara, Chikara Ohyama, Norihiko Tsuchiya, Tomonori Habuchi

**Affiliations:** ^1^ Department of Urology Akita University Graduate School of Medicine Akita Japan; ^2^ Department of Urology Yamagata University Faculty of Medicine Yamagata Japan; ^3^ Department of Urology Hirosaki University Graduate School of Medicine Hirosaki Japan; ^4^ Department of Urology Iwate Medical University Morioka Japan; ^5^ Department of Urology Fukushima Prefectural Medical University Fukushima Japan; ^6^ Department of Urology Aichi Cancer Center Nagoya Japan; ^7^ Department of Urology Tohoku University Graduate School of Medicine Sendai Japan; ^8^ Department of Urology and Andrology Tsukuba University Graduate School of Comprehensive Human Sciences Tsukuba Japan; ^9^ Department of Urology Miyagi Cancer Center Natori Japan

**Keywords:** axitinib, early response, metastatic renal cell carcinoma, VEGFR‐TKI

## Abstract

It remains unknown whether the early response to vascular endothelial growth factor receptor tyrosine kinase inhibitor (VEGFR‐TKI) management in malignancies links to long‐term survival. The objective of this study was to investigate the survival rates and predictive factors of early response in patients with metastatic renal cell carcinoma (mRCC) managed by VEGFR‐TKIs. From Jan. 2008 to Oct. 2018, 496 patients were treated with VEGFR‐TKIs as first‐line treatment at the eight Japanese hospitals (Michinoku RCC). Early cessation was defined as VEGFR‐TKIs being given up within 3 months after their initiation. The number of patients in early cessation VEGFR‐TKIs (Cohort I) was 173 (34.9%), and in long‐term use (Cohort II) was 323 (65.1%). The cancer‐specific survival (CSS) and overall survival (OS) were better in Cohort II. IMDC Poor‐risk was at risk of early cessation of a first‐line VEGFR‐TKI. Axitinib was the most preferred drug for long‐term treatment. On closer examination, both Cohort I and II were divided into two groups, the patients ceased VEGFR‐TKI due to adverse events (Group A [67 from Cohort I] and Group C [51 from Cohort II]) and disease progression (Group B [106 from Cohort I] and Group D [272 from Cohort II]). Despite that the cessation was adverse events, CSS and OS in Group A were worse than both Group C and D. Axitinib was administered with the safer profile. IMDC Poor risk was the risk factor for the early disease progression. Managing early adverse events may contribute to a better prognosis in mRCC patients treated VEGFR‐TKIs.

## Graphical abstract

Patients with early discontinuation of VEGFR‐TKIs had a worse survival rate, regardless of the cause of cessation. Axitinib was administered with a safe profile. Patients with IMDC poor risk was at risk for early disease progression. The quantification of response as early as three months of treatment allows a prognostic stratification.

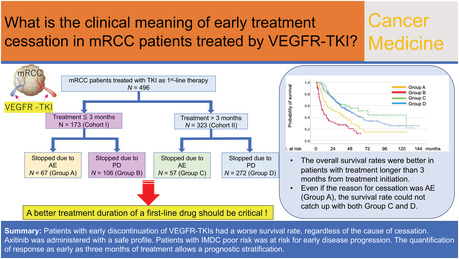

## 1 INTRODUCTION

Assessments of efficacy to vascular endothelial growth factor receptor (VEGFR) tyrosine kinase inhibitor (TKI) treatment in malignant neoplasms link to survival results. The analysis of response as early as 3 months on medication relates to a prognostic stratification. In patients with chronic myeloid leukemia, the depth of the efficacy as early as 3 months from the initiation of imatinib treatment has been proposed to correlate with a better survival outcome for nearly a decade.^1^ On the other hand, the implication of early response in metastatic renal cell carcinoma (mRCC) has never been evaluated yet. With competing TKI regimens and immune checkpoint inhibitors available, a timely conversion of management can be considered if an unfavorable outcome is expected due to early response failure.

Looking back to a pivotal phase III study of VEGFR‐TKIs, sunitinib showed a steep down in PFS 3 months after initiation^2^ and found the same results at 3 months in the sunitinib treatment group in recent clinical trials as a control drug.^3^, ^4^, ^5^, ^6^, ^7^ Almost 20–30% of patients gave up sunitinib 3 months after treatment initiation. Patients who showed durable efficacy from a first‐line VEGFR‐TKI would be expected a worthy outcome even from second‐ or late‐line VEGRF‐TKIs.^8^, ^9^, ^10^, ^11^ However, patients who gave up VEGFR‐TKIs because of progressive disease or adverse event (AE) might not have benefited from later VEGFR‐TKIs.^12^ If administrating VEGFR‐TKIs to the patient is not expected beneficial, it would waste the limited life of patients. Indeed, knowing about treatment prospects should be crucial to compose a treatment strategy for mRCC while nearly 10 regimes are available as a treatment option.

The objective of this study was to investigate the survival rates and predictive factors of cessation within 3 months in Japanese patients with mRCC treated with VEGFR‐TKIs as first‐line therapy.

## 2 MATERIAL AND METHODS

### 2.1 Patients

From Jan 2008 to Oct 2018, 703 mRCC patients were managed and registered for our study from the eight Japanese hospitals (Michinoku RCC). Of those patients, 496 patients were treated with VEGFR‐TKIs as first‐line treatment. Early cessation was defined as TKI‐VEGFR being given up within 3 months after its initiation (Cohort I). Patients who were treated with TKI‐VEGFR over 3 months were defined as Cohort II. For the purpose of closer analysis, each cohort was divided into two groups, respectively. The patients gave up VEGFR‐TKI because of adverse events (Group A and C) or disease progression (Group B and D) (Figure S1). The survival rates between cohorts or groups were compared.

### 2.2 Eligibility criteria

All included patients were proven histologically with mRCC in spite of Eastern Cooperative Oncology Group (ECOG) performance status (PS). This study was approved by all eight institutional review boards. All procedures were performed following the ethical standards laid down in the 1964 Declaration of Helsinki.

### 2.3 Objective

The primary purpose was to identify a clinical implication of early cessation of EGFR‐TKIs as first‐line therapy among mRCC patients by comparison of survival rates including cancer‐specific survival (CSS) and overall survival (OS). Then, the patients were classified into two cohorts depending on their reason for giving up treatment, and the survival rate was analyzed.

### 2.4 Treatment and follow‐up examinations

The following agreements were made before initiating medication and repeated during treatment based on the decision by each physician: entire medical history, physical checkup, ECOG PS, blood cell counts, biochemical profile (including electrolytes, kidney and liver function, coagulation, pancreatic amylase, and lipase), urinalyses, and radiography. Three simple candidates of biomarkers (neutrophil–lymphocyte ratio,^13^ C‐reactive protein,^14^ and alkaline phosphatase^15^) as predictive markers were analyzed. Tumor response was evaluated using the Response Evaluation Criteria in Solid Tumors guidelines version 1.1.

### 2.5 Statistical analysis

CSS was defined as the time between the initiation of VEGFR‐TKI treatment and  death because of progressive disease (PD). OS was defined as the time from VEGFR‐TKI start to death from any cause. The database record was closed upon patient death or the final follow‐up. Data were expressed as the median and interquartile range (IQR), and a p‐value less than 0.05 hac to be considered significant statistically. The chi‐square test was used to estimate the odds ratio (OR) in proportions on categorical factors. The survival curves were visualized by the Kaplan–Meier method to compare CSS and OS. The Cox proportional hazard regression analysis was applied for the investigation of hazard ratio (HR) and 95% confidence interval (CI). Multivariable analyses were performed by a logistic regression analysis. Clinical variables were included in the multivariable analysis if their univariate P‐value was less than 0.05. All data were analyzed by using SPSS version 26.0 statistical software (SPSS Japan Inc., Tokyo, Japan).

### 2.6 Human participants and Informed consent

Research involving human participants was supervised and approved by the Ethics board of the Akita University Hospital (Approved No. 2265) and the other participating institutes. All the patients in this study gave informed consent to participate. Since this study was a retrospective chart review analysis, we did not register this on website as clinical trial.

## 3 RESULTS

### 3.1 Patient characteristics

This study enrolled 703 patients who were diagnosed with advanced RCC and treated with systemic therapies at eight institutes from January 2008 to August 2018 (Michinoku RCC database). Of those, 496 patients who took VEGFR‐TKIs as first‐line therapy were investigated. The median patient age was 66 (IQR: 59–72) years. All patients were Japanese, and the cohort included 365 (73.6%) men and 131 (26.4%) women. The number of patients in early cessation of VEGFR‐TKIs within 3 months after initiation (Cohort I) was 173 (34.9%) and in longer treatment than 3 months (Cohort II) was 323 (65.1%) (Table 1). As first‐line systemic therapy, 272 patients were given sunitinib, 134 axitinib, 61 sorafenib, and 29 pazopanib. The patient's characteristics of the two groups were compared (Table 1). In Cohort I, more patients were stopped therapy due to adverse events, not the majority received a nephrectomy, with non‐clear cell histology, female gender, and higher risk classification per the International Metastatic Renal Cell Carcinoma Database Consortium (IMDC) than Cohort II (Table 1). On the other hand, the patients with pancreatic metastasis who were treated with axitinib achieved a long treatment duration (Table 1). Analyzed biomarkers, level of neutrophil–lymphocyte ratio, c‐reactive protein, and alkaline phosphatase were high in Cohort I (Table 1). The multivariable analysis resulted in IMDC Poor risk (OR, 1.960; 95% CI 1.140–3.447; *p* = 0.020) as a risk factor for early treatment failure (Table 2). In addition, patients treated with axitinib showed quite longer survival rate (OR, 0.311; 95% CI 0.176–0.550; *p* < 0.001) (Table 2).

**TABLE 1:** Patients characteristics of all cases and each cohort

	All patients	Cohort I	Cohort II	*p*
	(*N* = 496)	(*N* = 173)	(*N* = 323)	
Age				
Median year (IQR)	66 (59–72)	66 (59–73)	65 (59–72)	0.546
BMI				
Median kg/m^2^ (IQR)	22.6 (20.4–25.0)	22.0 (20.1–24.8)	23.0 (20.8–25.2)	0.065
Sex (%)				
Male	365 (74)	116 (67)	249 (77)	0.019
Female	131 (26)	57 (33)	74 (23)	
Reason of cessation (%)				
Disease progression	375 (76)	106 (61)	269 (83)	<0.001
Adverse events	118 (24)	67 (39)	51 (17)	
Drug (%)				
Sunitinib	272 (55)	111 (64)	161 (50)	0.004
Sorafenib	61 (12)	20 (12)	41 (13)	
Axitinib	134 (27)	32 (18)	102 (31)	
Pazopanib	29 (6)	10 (6)	19 (6)	
Nephrectomy (%)				
Yes	337 (68)	96 (55)	241 (75)	<0.001
No	159 (32)	77 (45)	82 (25)	
Histology (%)				
Clear cell	386 (78)	117 (68)	269 (83)	<0.001
With spindle component	68 (14)	28 (16)	40 (12)	
Papillary	20 (4)	9 (5)	11 (3)	
Others	40 (8)	18 (10)	22 (7)	
Unknown	50 (10)	29 (17)	21 (7)	
Grade (%)				
1	7 (2)	2 (1)	5 (2)	0.173
2	140 (28)	37 (21)	103 (32)	
3	189 (38)	64 (37)	125 (39)	
Unknown	160 (32)	70 (41)	90 (27)	
Clinical stage (%)				
1	57 (11)	17 (10)	40 (12)	0.380
2	37 (7)	10 (6)	27 (9)	
3	77 (16)	25 (14)	52 (16)	
4	311 (63)	118 (68)	193 (60)	
Unknown	14 (3)	3 (2)	11 (3)	
IMDC risk classification (%)				
Favorable	37 (7)	8 (5)	29 (9)	<0.001
Intermediate	230 (46)	64 (37)	166 (51)	
Poor	165 (34)	81 (47)	84 (26)	
Unclassified	64 (13)	20 (11)	44 (14)	
Metastatic site (%)				
1	214 (43)	67 (38)	147 (46)	0.053
2	157 (32)	53 (31)	104 (32)	
3≤	125 (25)	53 (31)	72 (22)	
Metastatic organ (%)				
Lung	302 (61)	99 (57)	203 (63)	0.243
Extra regional lymph node	171 (34)	70 (40)	101 (31)	0.037
Bone	160 (32)	65 (38)	95 (29)	0.068
Liver	66 (13)	30 (17)	36 (11)	0.053
Adrenal	48 (10)	20 (12)	28 (9)	0.338
Brain	32 (6)	15 (9)	17 (5)	0.178
Pancreas	27 (5)	2 (1)	25 (8)	0.001
CRP				
Median mg/L (IQR)	0.7 (0.2–4.3)	1.9 (0.3–7.0)	0.4 (0.1–2.7)	<0.001
NLR				
Median (IQR)	3.0 (2.1–4.5)	3.5 (2.5–5.8)	2.8 (1.9–4.1)	<0.001
ALP				
Median IU/mL (IQR)	271 (213–352)	282 (229–399)	262 (208–342)	0.049

Abbreviations: ALP, alkaline phosphatase; Cohort I, patients with treatment duration 3 months or less; Cohort II, patients with treatment duration over 3 months; CRP, C‐reactive protein; IMDC, International Metastatic Renal Cell Carcinoma Database Consortium; IQR, interquartile range; NLR, neutrophil–lymphocyte ratio.

**TABLE 2:** Predictive clinical valuable for early response to VEGFR‐TKIs in patients with metastatic renal cell carcinoma analyzed by using univariate and multivariable Logistic regression models

Risk factor	Risk category	Univariate				Multivariable			
		OR	95% CI		*p*	OR	95% CI		*p*
			Lower	Upper			Lower	Upper	
Gender	Male	1.653	1.098	2.488	0.016	0.818	0.458	1.459	0.495
Nephrectomy	not performed	2.358	1.595	3.484	<0.001	1.653	0.956	2.857	0.072
Histology	non‐clear cell	2.387	3.676	1.548	<0.001	1.416	0.782	2.564	0.251
**IMDC risk classification**	**poor**	**2.612**	**1.737**	**3.926**	**<0.001**	**1.960**	**1.114**	**3.447**	**0.020**
CRP	0.7 or more	2.673	1.804	3.958	<0.001	1.577	0.875	2.843	0.129
NLR	3.0 or more	1.770	1.190	2.631	0.005	1.151	0.675	1.962	0.605
ALP	271 or more	1.263	1.002	1.920	0.049	0.685	0.396	1.186	0.177
**Drug**	**Axitinib**	**0.492**	**0.314**	**0.771**	**0.002**	**0.311**	**0.176**	**0.550**	**< 0.001**
Metastatic organ	Pancreas	0.140	0.033	0.598	0.008	0.280	0.058	1.346	0.112
	Extra regional LN	1.511	1.027	2.223	0.036	1.303	0.781	2.174	0.310

Factors with statistically significant are indicated in bold.

Abbreviations: ALP, alkaline phosphatase; CI, confidence interval; CRP, C‐reactive protein; IMDC, International Metastatic Renal Cell Carcinoma Database Consortium; LN, lymph node; NLR, neutrophil–lymphocyte ratio; OR, odds ratio; TKI, tyrosine kinase inhibitor; VEGFR, vascular endothelial growth factor receptor.

### 3.2 Antitumor effects

**FIGURE 1:**
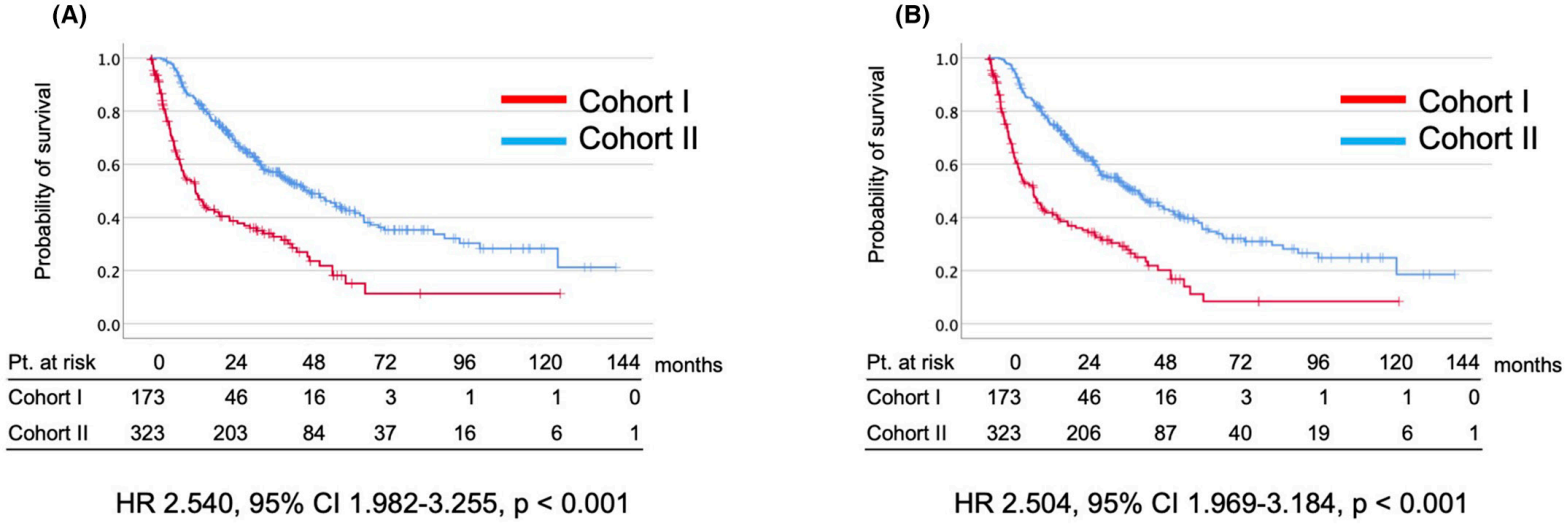
Kaplan–Meier curve of cause‐specific survival (A) and overall survival (B) between early ceased patients (Cohort I) and longer treated patients (Cohort II) in mRCC patients treated with VEGFR‐TKIs as the first‐line treatment in all cases. The median CSS and OS were 14.3 and 14.3 months in Cohort I and 49.9 and 47.1 months in Cohort II, respectively

In all cases, the median CSS and OS were 14.3 and 14.3 months in Cohort I and 49.9 and 47.1 months in Cohort II, respectively (Figure 1). In comparative analysis, CSS (HR 2.540, 95% CI 1.982–3.255, *p* < 0.001) and OS (HR 2.504, 95% CI 1.969–3.184, *p* < 0.001) were significantly better in Cohort II (Figure 1). IMDC poor‐risk (OR, 1.960; 95% CI 1.114–3.447; *p* = 0.020) was the risk factor for the early failure of first‐line VEGFR‐TKIs (Table 2). The patients managed with axitinib as first‐line therapy achieved a significantly longer treatment duration (OR, 0.311; 95% CI 0.176–0.550; *p* < 0.001) (Table 2).

### 3.3 Subgroup analysis

For the purpose of closer analysis, we divided the cohorts into two groups each, the patients gave up VEGFR‐TKI because of adverse events (Group A and C) or disease progression (Group B and D) (Table 3).

**TABLE 3:** Characteristics in each group

	All patients	Group A	Group B	Group C	Group D
	(*N* = 496)	(*N* = 67)	(*N* = 106)	(*N* = 51)	(*N* = 272)
Age					
Median year (IQR)	68 (62–75)	68 (62–74)	65 (59–71)	69 (63–75)	65 (58–72)
BMI					
Median kg/m^2^ (IQR)	22.4 (20.9–25.1)	22.0 (20.8–25.0)	22.2 (19.8–24.6)	23.0 (21.1–25.4)	23.0 (20.4–25.2)
Sex (%)					
Male	86 (73)	50 (75)	66 (62)	36 (71)	213 (78)
Female	32 (27)	17 (25)	40 (38)	15 (29)	59 (22)
Drug (%)					
Sunitinib	69 (58)	49 (73)	62 (58)	20 (39)	141 (52)
Sorafenib	8 (7)	5 (7)	15 (14)	3 (6)	38 (14)
Axitinib	31 (26)	7 (11)	25 (24)	24 (47)	78 (29)
Pazopanib	10 (8)	6 (9)	4 (4)	4 (8)	15 (6)
Nephrectomy (%)					
Yes	82 (69)	45 (67)	51 (48)	37 (73)	204 (75)
No	36 (31)	22 (33)	55 (52)	14 (27)	68 (25)
Histology (%)					
Clear cell	99 (84)	52 (78)	65 (61)	47 (92)	222 (89)
With spindle component	14 (12)	7 (10)	21 (20)	7 (14)	33 (12)
Papillary	3 (3)	2 (3)	7 (7)	1 (2)	10 (4)
Others	4 (3)	4 (6)	14 (13)	0 (0)	21 (8)
Unknown	12 (10)	9 (13)	20 (19)	3 (6)	19 (7)
Grade (%)					
1	2 (2)	1 (2)	1 (1)	1 (2)	4 (1)
2	38 (32)	21 (31)	16 (15)	17 (33)	86 (32)
3	43 (36)	21 (31)	43 (41)	22 (43)	103 (38)
Unknown	35 (30)	24 (36)	46 (43)	11 (21)	79 (29)
Clinical stage (%)					
1	11 (9)	6 (9)	11 (10)	5 (10)	35 (13)
2	9 (8)	7 (11)	3 (3)	2 (4)	25 (9)
3	20 (17)	11 (16)	14 (13)	9 (18)	43 (16)
4	76 (64)	41 (61)	77 (73)	35 (68)	158 (58)
Unknown	2 (2)	2 (3)	1 (1)	0 (0)	11 (4)
IMDC risk classification (%)					
Favorable	7 (6)	4 (6)	4 (4)	3 (6)	26 (9)
Intermediate	57 (48)	30 (45)	34 (32)	27 (53)	139 (51)
Poor	43 (37)	26 (39)	55 (52)	17 (33)	67 (25)
Unclassified	11 (9)	7 (10)	13 (12)	4 (8)	40 (15)
Metastatic site (%)					
1	56 (47)	32 (48)	35 (33)	24 (47)	123 (45)
2	35 (30)	19 (28)	34 (32)	16 (31)	88 (32)
3≤	27 (23)	16 (24)	37 (35)	11 (22)	61 (23)
Metastatic organ (%)					
Lung	66 (56)	34 (51)	65 (61)	32 (63)	171 (63)
Extra regional lymph node	36 (31)	22 (33)	48 (45)	14 (27)	87 (32)
Bone	42 (36)	26 (39)	39 (37)	16 (31)	79 (29)
Liver	14 (12)	8 (12)	22 (21)	6 (12)	30 (11)
Adrenal	9 (8)	6 (9)	14 (13)	3 (6)	25 (9)
Brain	6 (5)	5 (7)	10 (9)	1 (2)	16 (6)
Pancreas	6 (5)	2 (3)	0 (0)	4 (8)	21 (8)
CRP					
Median mg/L (IQR)	0.6 (0.1–3.6)	1.0 (0.2–3.8)	2.5 (0.3–8.1)	0.4 (0.1–3.3)	0.4 (0.1–2.6)
NLR					
Median (IQR)	3.0 (2.1–5.1)	3.4 (2.1–5.6)	3.5 (2.6–5.8)	2.6 (1.7–4.1)	2.8 (1.9–4.1)
ALP					
Median IU/ml (IQR)	267 (217–371)	285 (228–425)	276 (229–359)	259 (201–305)	266 (209–345)

Abbreviations: ALP, alkaline phosphatase; BMI, body mass index; CRP, C‐reactive protein; Group A, patients discontinued treatment within 3 months due to adverse event; Group B, patients discontinued treatment within 3 months due to disease progression; Group C, patients gave up treatment 3 months after initiation due to adverse event; Group D, patients gave up treatment 3 months after initiation due to disease progression; IMDC, International Metastatic Renal Cell Carcinoma Database Consortium; IQR, interquartile range; NLR, neutrophil–lymphocyte ratio; TKI, tyrosine kinase inhibitor; VEGFR, vascular endothelial growth factor receptor.

#### 3.3.1 Group A versus Group C (Treatment was ceased by AE)

**FIGURE 2:**
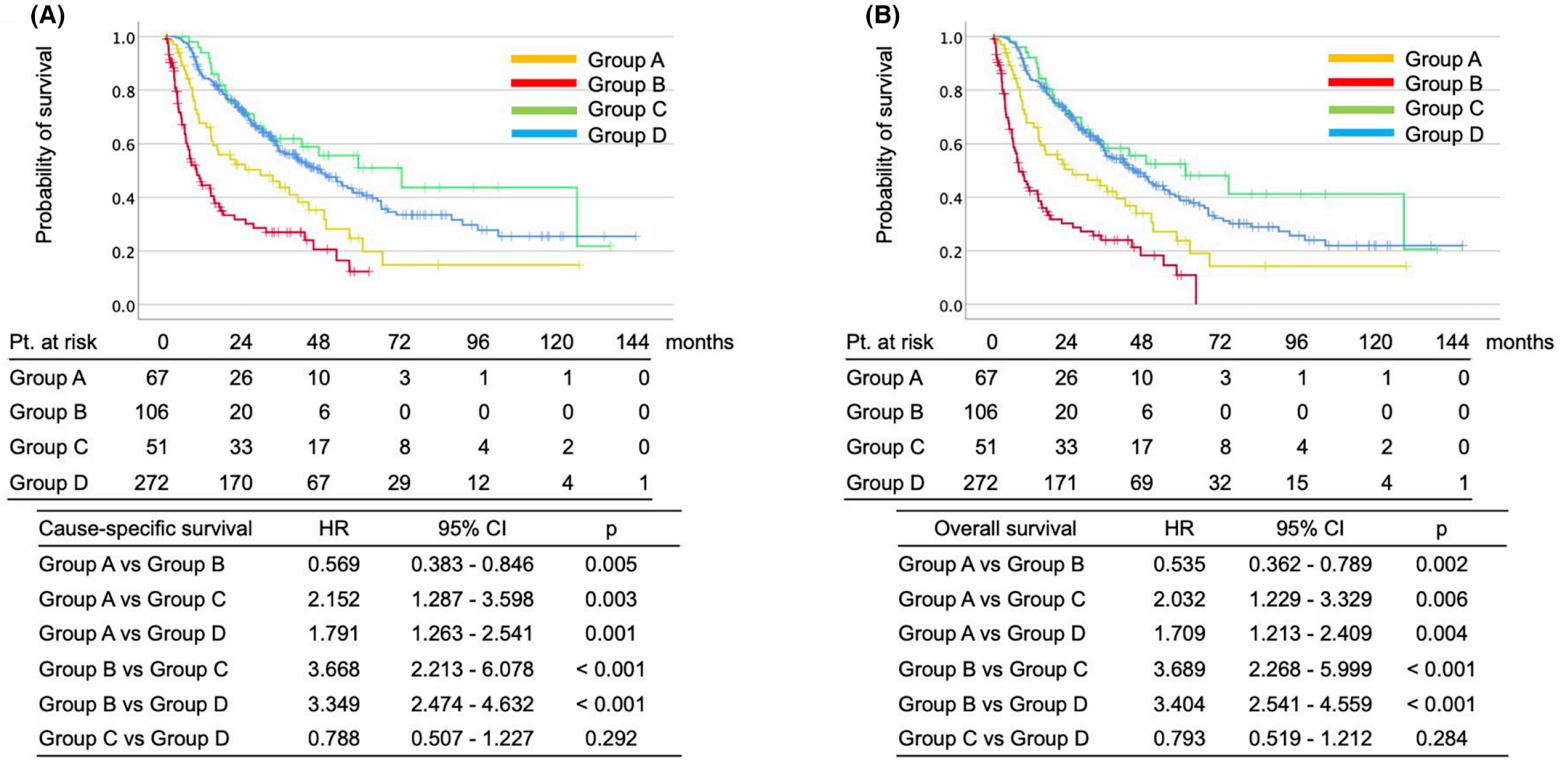
Kaplan–Meier curve of cause‐specific survival (A) and overall survival (B) among Group A (patients discontinued treatment within 3 months due to adverse event), Group B (patients discontinued treatment within 3 months due to disease progression), Group C (patients gave up treatment 3 months after initiation due to adverse event), and Group D (patients gave up treatment 3 months after initiation due to disease progression) in mRCC patients

(Table S1) CSS (HR 2.152, 95% CI 1.287–3.598, *p* = 0.003) and OS (HR 2.023, 95% CI 1.229–3.329, *p* = 0.006) were poor in group A (Figure 2). However, both median survival periods were enough long (CSS 29.7 months and OS 25.0 months in group A; CSS 73.7 months and 60.2 months in group C) (Figure 2).

#### 3.3.2 Group B versus Group D (Treatment was ceased by PD)

(Table S2) It was natural that CSS (HR 3.349, 95% CI 2.474–4.632, *p* < 0.001) and OS (HR 3.404, 95% CI 2.541–4.559, *p* < 0.001) were quite poor in group B (Figure 2). Median survival periods were CSS 9.9 months and OS 8.3 months in group B, and CSS 48.6 months and 44.3 months in group D (Figure 2).

#### 3.3.3 Group A (Treatment was ceased by AE in Cohort I) versus Group D (Treatment was ceased by PD in Cohort II)

(Table S3) We also would like to know whether the patients who ceased treatment within 3 months by AE could catch up survival rate with the patients who were treated for a long period. CSS (HR 2.152, 95% CI 1.287–3.598, *p* = 0.003) and OS (HR 2.023, 95% CI 1.229–3.329, *p* = 0.006) were poor in group A (Figure 2).

## 4 DISCUSSION

Patients with early failure of first‐line VEGFR‐TKI showed a worse survival rate than patients with a longer response not depending on the reasons for cessation. Axitinib was more safely given than other VEGFR‐TKIs and contributed to a better treatment duration as first‐line treatment. IMDC poor risk was strongly related to early cessation of first‐line VEGFR‐TKI treatment. After dividing the patients into the four groups by the treatment duration and the reason for cessation, CSS and OS were the better in the long‐term treated Groups and the poorest in the early ceased treatment due to PD. In addition, despite the cessation due to AEs, the CSS and OS in Group A were worse than in the long‐term treatment Groups (both Group C and D).

A better treatment duration of a first‐line drug should be critical for managing mRCC patients.^16^ Patients who gave up the first‐line VEGFR‐TKI due to AE, the patients did not always experience AEs while even administrating other kinds of VEGFR‐TKI.^8^ However, a first‐line therapy must be crucial to achieving better clinical outcomes.^17^ In fact, intolerable drugs would waste a lifetime as worse as vigor directly.^18^, ^19^ Our results could suggest that axitinib might be a better choice because of its manageable nature.^20^, ^21^ This manageable character of axitinib might relate to the higher relative dose intensity than the other VEGFR‐TKIs. A significantly higher rate of toxicity‐related treatment discontinuation by VEGFR‐TKIs except axitinib was reported by several authors.^20^ These discontinuations could cause undesirable treatment suspensions.^22^ Treatment interruption of sunitinib showed a worse survival rate in mRCC patients.^22^ Moreover, the aftereffect of sunitinib toxicities is an non‐negligible factor led to poor results.^19^ Because the recommended sunitinib dose would be intolerable, some physicians try to solve it by primary dose reduction^16^ or/and modifying the schedule.^23^

The patients who occurred early treatment failure due to disease progression resulted in a worse survival rate in our cohorts. Such a patient population should be administrated IO combination therapy as a first‐line therapy instead of VEGR‐TKIs. Patients with an IMDC poor‐risk are thought to be the impractical candidate for VEGFR‐TKIs.^3^ Without argument, nivolumab and ipilimumab therapy should be a better treatment for this population.^24^ From the other aspect, primary resistance at first‐line VEGFR‐TKI was a potential predictor as not suitable for more VEGFR‐TKIs.^10^, ^11^ The high‐grade tumor should also be highly resistant to VEGFR‐TKIs.^25^, ^26^ The high stage being at the time of the first diagnosis, VEGFR‐TKIs should be also less effective even in the IO era.^27^ In patients with these three factors, IO combination therapy might be a better treatment.

This study contains several potential sources of bias that should be noted as limitations. First, we were not able to get rid of selection bias and other unexpected confounders due to the retrospective nature of the study. Second, medical information about adverse events was not gathered in the cohort. Third, consequences of our study may not be generalized to other patient's populations due to differences in medical practices or health insurance. However, this is the first study to report the clinical significance of early response of VEGFR‐TKIs as first‐line therapy and confirmed their risk factors. Further research is needed to address the meaning of early cessation in patients with mRCC.

## 5 CONCLUSIONS

The patients with the early cessation of VEGFR‐TKIs showed a worse survival rate without regard to the cause of cessation. Axitinib was used longer than the other VEGFR‐TKIs with a safer profile. The IMDC Poor risk was the risk factor for the early treatment failure due to disease progression. Careful handling of early adverse events may contribute to a better prognosis in mRCC patients treated VEGFR‐TKIs as first‐line therapy.

## AUTHOR CONTRIBUTIONS

**Ryuta Sobu:** Data curation (equal); formal analysis (equal); writing – original draft (equal). **Kazuyuki Numakura:** Conceptualization (lead); data curation (lead); formal analysis (lead); funding acquisition (lead); project administration (lead); supervision (lead); visualization (lead); writing – original draft (lead); writing – review and editing (lead). **Sei Naito:** Data curation (equal); project administration (equal); writing – review and editing (equal). **Shingo Hatakeyama:** Data curation (equal); validation (equal); writing – review and editing (lead). **Renpei Kato:** Data curation (equal); writing – review and editing (equal). **Tomoyuki Koguchi:** Data curation (equal); writing – review and editing (equal). **Takahiro Kojima:** Data curation (equal); writing – review and editing (equal). **Yoshihide Kawasaki:** Data curation (equal); writing – review and editing (equal). **Shuya Kandori:** Data curation (equal); writing – review and editing (equal). **Sadafumi Kawamura:** Data curation (equal); writing – review and editing (equal). **Yoichi Arai:** Data curation (equal); writing – review and editing (equal). **Akihiro Ito:** Data curation (equal); writing – review and editing (equal). **Hiroyuki Nishiyama:** Data curation (equal); writing – review and editing (equal). **Yoshiyuki Kojima:** Data curation (equal); writing – review and editing (equal). **Wataru Obara:** Data curation (equal); writing – review and editing (equal). **Chikara Ohyama:** Data curation (equal); writing – review and editing (equal). **Norihiko Tsuchiya:** Data curation (equal); supervision (equal); writing – review and editing (equal). **Tomonori Habuchi:** Funding acquisition (lead); supervision (lead); visualization (lead); writing – original draft (equal); writing – review and editing (lead).

## FUNDING INFORMATION

This study was supported by Grants‐in‐Aid for Scientific Research, Japan (Grant No.: 17 K11121 and 20 K09553).

## CONFLICT OF INTEREST

Dr. Habuchi received honoraria from Novartis Pharma, Pfizer Co., GlaxoSmithKline, and Ono Pharma Co.

Other authors have no conflicts of interest.

## Supporting information


Figure S1

Table S1

Table S2

Table S3

## Data Availability

All data are available for editors and reviewers by an appropriate reason.
