# Supplementary material for: Clinical impact of early response to first‐line VEGFR‐TKI in patients with metastatic renal cell carcinoma on survival: A multi‐institutional retrospective study
Source: Cancer Med. 2022 Oct 6;12(4):4100–9. doi: 10.1002/cam4.5268 (PMC9972009; doi:10.1002/cam4.5268)
Supplement: Supplementary file 1 — Figure S1 [file CAM4-12-4100-s004.pdf]

Supplement Figure 1. Selection of patients. Of 703 patients with mRCC, we selected 496 patients who were treated with VEGFR-TKIs as first-line in this study.

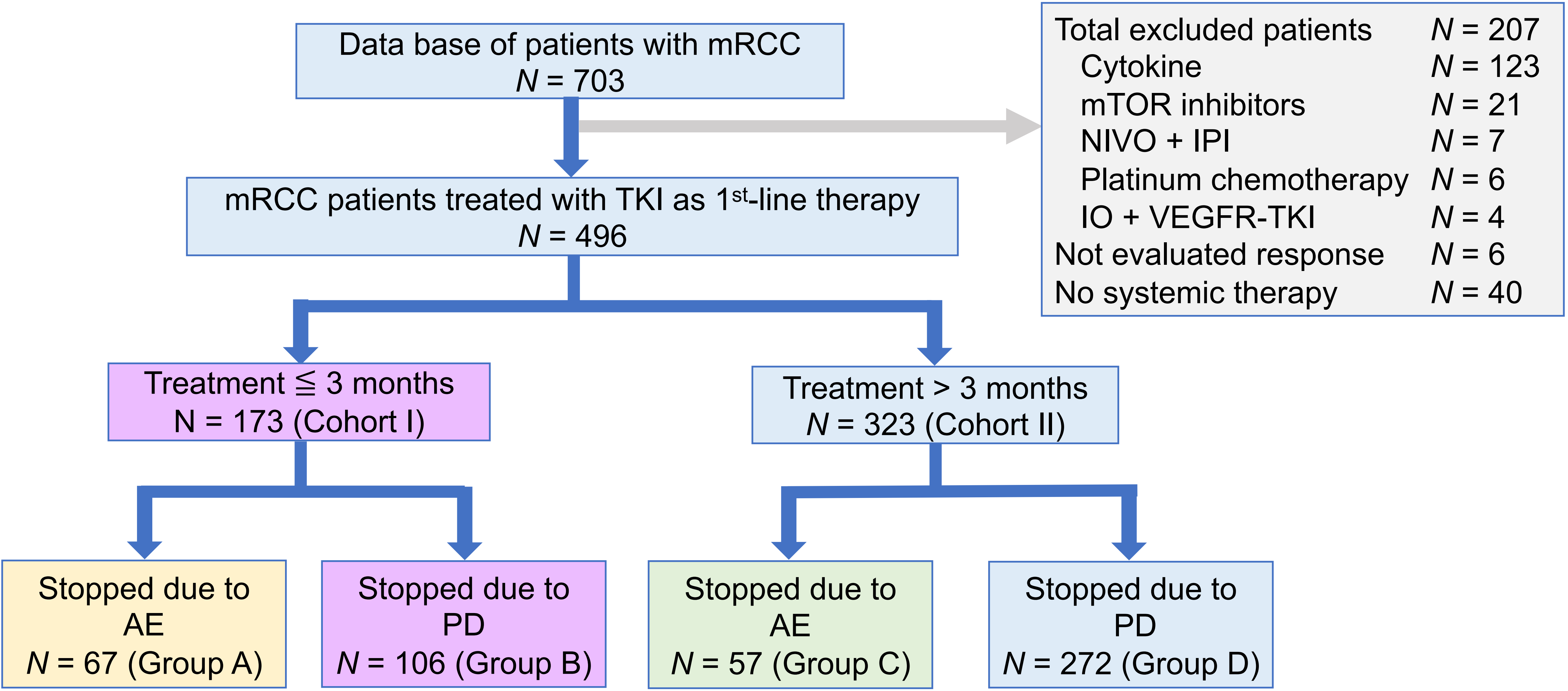

mRCC, metastatic renal cell carcinoma; VEGFR, vascular endothelial growth factor receptor; TKI, tyrosine kinase inhibitor; mammalian target of rapamycin; NIVO, nivolumab; IPI, ipilimumab, IO, immuno-oncology drug
